# The emerging role of the gut mycobiome in liver diseases

**DOI:** 10.1080/19490976.2023.2211922

**Published:** 2023-05-15

**Authors:** Natalia Szóstak, Marek Figlerowicz, Anna Philips

**Affiliations:** Institute of Bioorganic Chemistry, Polish Academy of Sciences, Poznan, Poland

**Keywords:** Gut microbiome, mycobiome, liver diseases, alcohol-associated liver disease, nonalcoholic fatty liver disease, primary sclerosis cholangitis, cirrhosis, hepatocellular carcinoma

## Abstract

In recent years, it has become clear that gut microbiota plays a major role in the human body, both in health and disease. Because of that, the gut microbiome and its impact on human well-being are getting wider and wider attention. Studies focused on the liver are not an exception. However, the majority of the analyses are concentrated on the bacterial part of the gut microbiota, while the fungi living in the human intestines are often omitted or underappreciated. This review is focused on the gut mycobiome as an important factor that should be taken into consideration regarding liver homeostasis and its perturbations. We have collected the findings in this field and we discuss their importance. We aim to emphasize the fungal compositional changes related to liver diseases and, by that, provide novel insights into the directions of liver research and gut microbiota as a therapeutic target for liver diseases.

## Introduction

The human gut harbors a flourishing variety of organisms belonging to all kingdoms of life.^1^ The most numerous and well-studied are bacteria, but the others, less abundant, such as archaea, viruses, protozoa, and fungi, also require attention. In recent years, with the advent of metagenomics studies, it has become clear that this complex internal ecosystem called the gut microbiota is not just a by-passenger but is of extreme importance to human health and maintaining proper homeostasis. Disruption of the normal gut microbiota can have serious health consequences and lead to the development or acceleration of disease progression.^2^ Noticeably, this concerns not only the pathophysiology of intestinal but also extraintestinal diseases. Taking this into account, it is not surprising that the gut microbiome and its impact on human well-being are getting wider and wider attention.

The human gut microbiota has been extensively studied in relation to many host factors such as age^3,4^, gender^5,6^, diet^7,8^, lifestyle^9,10^, and diseases, such as obesity^11–13^, diabetes^14,15^, rheumatoid arthritis^16,17^, autism spectrum disorder^18–20^, and a variety of cancers^21,22^. Liver diseases are also on the list, which is not surprising, taking into account the global burden of liver diseases, accounting for approximately 2 million deaths each year.^23^ Recent interest in the relationship between liver disease and gut microbiota revealed that gut microbiota structure changes in various liver diseases, and there is a crosstalk between gut microbiota and the liver.^24,25^ The bidirectional communication between them is provided by the biliary system and the portal vein widely known as the gut-liver axis.^26^

One of the most well-known hypotheses related to the role of the microbiome in liver health and diseases is the so-called leaky gut hypothesis. It assumes that the increased intestinal permeability resulting from an inflamed gut causes the translocation of microbes or microbial components and products (e.g., toxins) to the portal-venous system, further leading to harmful liver inflammation or directly damaging liver structure.^27,28^

So far, most research, including liver diseases, has focused on the bacterial part of the gut microbiota. Only recently, it has been recognized that gut fungi, known as mycobiome, are also essential members of the gut microbiota.^29–31^ This fact might seem astonishing, as intestinal fungi constitute only less than 1% of the species that can be found in the human gut.^32^ Yet, the researchers estimate that of the~10^13^ microorganisms in the human gastrointestinal tract^33^, about a billion compose the gut mycobiota.^34^ The majority of them belong to the *Ascomycota* and *Basidiomycota* phyla^35,36^, and *Saccharomyces*, *Candida*, and *Malassezia* are among the most abundant fungal genera, probably constituting the core mycobiota.^35,37,38^ Like intestinal bacteria, some gut fungi are beneficial, while others may be harmful. The latter also concerns fungi commonly found in the human gut, e.g., *Candida albicans*, an opportunistic pathogen that, when out of control, may cause deadly candidiasis.^39,40^

Regarding the emerging role of the gut mycobiome in liver diseases, this review is focused on the gut mycobiome as a vital factor for maintaining liver homeostasis and understanding liver disease pathogenesis. Our goal is to highlight the compositional changes associated with liver diseases and, by that, provide novel insights into the directions of liver research and gut microbiota as a therapeutic target for liver diseases. We also provide a summary of the alterations of mycobiome in liver diseases (Table 1).

**Table 1.** Summary of the alterations of mycobiome in liver diseases.

Liver disease	Mycobiome alterations	References
Ethanol-induced liver disease	Fungal richness ↑ Fungi diversity ↑ *Humicola, Fusarium, Aspergillus* ↑ *Candida* ↓ *Candida parapsilosis* ↓ 1,3-β-glucan plasma level ↑ Antifungal drugs alleviated the symptoms of ethanol-induced liver disease. ↓	44(M)
ALD	Fungal richness ↓ Fungal diversity ↓	50,51
	*Candida*, *Debaryomyces, Pichia*, *Kluyveromyces*, *Issatchenkia*, *Scopulariopsis*↑ *Candida zeylanoides*, *Issatchenkia orientalis*, *Scopulariopsis cordiae*↑ *Malassezia* in AUD with the progressive liver disease ↑ *Aspergillus* ↓ *Kazachstania humilis* ↓ Anti-*C. albicans* immunoglobulin G (IgG) and M (IgM) serum levels ↑	49
	*Candida albicans*↑	44,49–51
	Candidalysin directly cause hepatocyte death and liver injury.	50
	*Meyerozyma guilliermondii*↑ Inoculation of *M. guilliermondii* into fungi-free mice worsened the features of AHS. 1,3-β-glucan from *Wolfporia cocos* reduces hepatic inflammatory injury and steatosis.	48(M)
Obesity	Fungal diversity at a family level ↓	60
	*Ascomycota* (phylum) ↑ *Saccharomyces*, *Tremellomycetes* (class) ↑ *Dipodascaeae*, *Sachharomycetaceae* (family) ↑ *Candida*, *Nakaseomyces, Penicillium* ↑	11–13
	*Candida parapsilosis*↑	61(M)
	yeast ↑	13
	*Zygomycota* (phylum) ↓ *Mucor* (genus) ↓ Negative correlation reversed after weight loss. Increased with the occurrence of metabolic abnormalities: *Ascomycota* (phylum) ↑ *Saccharomycetes, Tremellomycetes, Cystobasidiomycetes* (class) ↑ *Erythrobasidiaceae, Dipodascaceae* (family) ↑ *Aspergillus, Eurotium, Rhodotorula* (genus) ↑ Positively correlated with protection from metabolic disorders: *Zygomycota* (phylum) ↑ *Agaricomycetes*, *Eurotiomycetes* (class) ↑ *Mucoraceae*, *Nectriaceae*, *Ceratocystidaceae*, *Corticiaceae*, *Debariomycetaceae*, *Hypocraceae* (family) ↑ *Mucor, Penicillium, Monilliela*, *Ceratocystis* ↑	60
	High-fat diet positively correlated with: *Alternaria*, *Saccharomyces*, *Septoriella*, *Tilletiopsis* ↑ *Saccharomyces* cerevisiae, Tilletiopsis *washingtonensis* ↑	62(M)
NAFLD	Talaromyces, Paraphaeosphaeria , *Lycoperdon*, *Curvularia*, *Phialemoniopsis* ,*Paraboeremia*, *Sarcinomyces*, *Cladophialophora*, *Sordaria* ↑ *Leptosphaeria*, Pseudopithomyces, Fusicolla ↓ fungal intrakingdom correlations ↑ Fungal richness ↑ (NASH) Fungal evenness ↑ (NASH) *Paramycosphaerella*, *Fusicolla*, *Arthrinium*, *Triparticalcar*, *Trichoderma*, and *Cladosporium* ↑ (NASH) *Cladosporium*, *Staphylotrichum*, *Paecilomyces*, and *Thermomyces* ↑ (fibrosis) *Pulvinula* ↓ (fibrosis, ALT, AST)	65
	*C. albicans*↑	66
	Treatment with antifungal amphotericin B reduced liver damage.	^66(M)^
PSC	serum ASCA ↑	73
	*Candida* in bile associated with reduced survival in PSC patients.	74
	*Sordariomycetes* (class) ↑ *Exophiala* ↑ *S. cerevisiae* ↓ ITS2/16S diversity ↑ Disruption in the bacteria-fungi correlation network	77
	*Candida*↑ *Humicola* (*H. grisea -* reclassified as *Trichocladium griseum*) ↑	79
	A systemic infection *Exophiala dermatitidis* can mimick PSC in a patient without immunodeficiency	81
	*E. dermatitidis* infection leads to an end-stage liver disease characterized by cholestasis and dilatation of intrahepatic bile ducts.	82
Cirrhosis	Fungal diversity ↓ Fungal diversity negatively corelated with MELD score. *Candida* ↑ *Candida albicans* ↑ *Bacteroidetes*/*Ascomycota* changes with cirrhosis severity ↓ *Bacteroidetes*/*Ascomycota* ratio was associated with lower hospitalizations	87
	Correlation with esophageal candidiasis	93
	4.4 times higher odds of mortality in cirrhosis patients with invasive candidiasis.	94
Cirrhosis due to HBV	fungal abundance ↑ fungal diversity ↑	89
	*Aspergillus*, *Candida*, *Galactomyces*, *Saccharomyces*, *Chaetomium*↑ *C. albicans*, *C. parapsilosis*, *C. krusei*, *C. parapsilosis*, *C. glabrata*, *C. tropicalis*, *S. cerevisiae*↑	90–92
HCC	aflatoxin B1 ↑	100,101
	The interaction between AFB1 and HBV infection increased the risk of HCC 60-fold.	104
	gut mycobiome diversity ↓ *Leotiomycetes* (class) ↓ *Myxotrichaceae*, Debaryomycetaceae, Trichomonascaceae, *Saccharomycetaceae* (family) ↓ *Saccharomycetales fam Incertae sedis* (family) ↑ *Kazachstania*, *Debaryomyces*, *Xeromyces*, Amorphotheca, Blastobotrys ↓ Candida ↑ *C. albicans* ↑	112

ALD – Alcohol-associated liver disease, ALT – Alanine aminotransferase, AST – Aspartate aminotransferase, ASCA – anti-*S. cerevisiae* antibodies, HBV – hepatitis B virus, HCC – Hepatocellular carcinoma, ITS – Interal transcribed spacer, NAFLD – Nonalcoholic fatty liver disease, NASH – Nonalcoholic steatohepatitis, PSC – Primary sclerosis cholangitis, (M) – studies on mouse.

## Alcohol-associated liver disease

Alcohol-associated liver disease (ALD) is a common disease caused by the overuse of alcohol that leads to liver steatosis, alcohol-associated hepatitis, cirrhosis, and potentially hepatocellular carcinoma (HCC).^41^ ALD is a severe global problem as it has been estimated that 60–80% of liver-related deaths can be attributed to excessive alcohol consumption. Although alcohol-mediated reactive oxygen species formation and hepatic inflammation are the main drivers of ALD development^42^, ALD has also been recently linked to the changes in the gut mycobiome structure. One of the studies showed that germ-free mice obtaining intestinal microbiota from patients with alcohol hepatitis had more severe liver inflammation and disruption of the intestinal integrity in response to alcohol. Contrary to that, mice receiving microbiota from patients without alcoholic hepatitis were able to reverse the alcohol-caused liver injuries.^43^ Even though bacteria had been the main point of interest, there are also some interesting reports regarding the gut mycobiota changes and the role of fungi in ALD.

Studies of mice under chronic ethanol administration showed overgrowth of intestinal fungi and increased plasma levels of β-glucan, the main component of the fungal cell walls.^44^ Reducing an increase in intestinal fungi by antifungal drugs alleviated the symptoms of ethanol-induced liver disease. The possible mechanism of liver damage by fungi includes induction of liver inflammation by β-glucan via the C-type lectin-like receptor CLEC7A on Kupffer cells and possibly other bone marrow-derived cells. This process leads to the upregulation of the inflammatory cytokine IL-1β^45^, which leads to hepatocyte damage and promotes the development of ethanol-induced liver disease. Moreover, it has been shown that blocking IL-1β prevents alcoholic steatohepatitis in mice^46^, suggesting possible routes for therapeutic intervention for attenuation of alcohol-related liver disease. However, water-insoluble polysaccharide, a 1,3-β-glucan from *Wolfporia cocos*, an edible mushroom used in Chinese traditional medicine for over 2000 years^47^, reduces the hepatic inflammatory injury and steatosis in mice with alcoholic hepatic steatosis.^48^ This observation suggests the protective effect of 1,3-β-glucan in liver diseases, but the mechanism for this action needs further study.

At the gut mycobiome compositional level, patients with alcohol use disorder (AUD) had a significantly increased abundance of the *Candida*, *Debaryomyces*, *Pichia*, *Kluyveromyces*, *Issatchenkia*, and *Scopulariopsis* genera, and *C. albicans*, *Candida zeylanoides*, *Issatchenkia orientalis*, and *Scopulariopsis cordiae* species compared with a control group.^49^ Notably, short abstinence periods of 2 weeks were associated with a significantly lower abundance of fungi belonging to *Candida*, *Malassezia*, *Pichia*, *Kluyveromyces*, *Issatchenkia*, *Claviceps*, *Cyberlindnera*, and *Hanseniaspora*, as well as *C. albicans*, *C. zeylanoides*, *I. orientalis*, and *Cyberlindnera jadinii*. These changes were accompanied by significantly higher specific anti-*C. albicans* immunoglobulin G (IgG) and M (IgM) serum levels in AUD subjects compared to control participants and significantly decreased anti-*C. albicans* IgG levels in the AUD group after two weeks of abstinence. Moreover, a higher level of *Malassezia* fungi was observed in AUD with the progressive liver disease compared with non-progressive liver disease. There were also some opposite changes, as an abundance of fungi belonging to the *Aspergillus* genus and *Kazachstania humilis* species was significantly decreased in patients with AUD relative to controls.

The overgrowth of *C. albicans* has been observed in ALD subjects compared with nonalcoholic controls also in other studies^44,50,51^, suggesting that there might be a real association between *C. albicans* and ALD. It has been shown that dysbiosis-related exotoxins, such as candidalysin from *C. albicans*, directly cause hepatocyte death and liver injury, and it is also associated with liver disease severity and mortality in patients with alcoholic hepatitis.^50^

Another study has shown the increase of the commensal fungus *Meyerozyma guilliermondii* in the feces of mice with alcoholic hepatic steatosis (AHS).^48^ Of notice, the inoculation of *M. guilliermondii* into fungi-free mice worsened the features of AHS. *M. guilliermondii* has been shown to generate prostaglandin E_2_ (PGE_2_) by biotransformation of arachidonic acid and the gut fungi (*M. guilliermondii*)-induced PGE_2_ production in the liver was confirmed as one of the mechanisms in the chronic AHS.

It should be emphasized that even considering the reported association between altered gut mycobiota in ALD, determining whether these changes are connected rather to ALD or chronic alcohol uptake is extremely hard. It is also hard to distinguish between the cause and the effects. After all, fungi are present in many types of alcohol as they are widely used in alcohol production, and the altered gut mycobiota in ALD subjects may be just the effect of alcohol consumption and may not be connected directly to ALD. For example, there is an association between the high prevalence of *Saccharomyces* and the consumption of yeast-containing foods such as beer and bread.^11^ Furthermore, even focusing on alcoholism, the mechanisms leading to gut mycobiota dysbiosis in chronic alcohol uptake are unclear. It is possible that fungi are a consequence of a bacterial imbalance in the gut and are simply taking over a niche usually colonized by bacteria. This possibility seems to be supported by the fact that fungal bloom often follows bacteria dysbiosis after antibiotic treatment.^52^ Answer to the question of why fungi from the *Candida* genus increase in abundance as a result of alcohol usage may also be relatively trivial, as fungi from this genus are also among the most abundant in the human gut^39^, meaning that they can also be the first and the best adapted to take advantage. Regardless of these hypotheses, further case-designed studies are necessary to confirm or deny the active role of the gut mycobiome in the ALD progression.

## Nonalcoholic fatty liver disease and obesity

Nonalcoholic fatty liver disease is one of the most important causes of liver disease worldwide – its prevalence is estimated to be 24–45%, with a predicted increasing trend.^53,54^ Moreover, it is also characterized by high mortality, accounting for 23% to 29% of total deaths.^55^ A growing body of evidence suggests that NAFLD is a multisystem disease and is often considered the liver manifestation of metabolic syndrome.^56^ NAFLD is also associated with obesity and type 2 diabetes. Although recent research indicates that gut microbiota is one of the factors that come into play in NAFLD^57,58^, the role of gut fungi is not well studied, and the majority of studies are focused rather on obesity than NAFLD itself.

Studies of lean and obese mice suggest that the gut microbiota affects the energy balance by influencing the efficiency of calorie harvest and how this energy is used and stored.^59^ Obesity was also significantly correlated with a decrease in fungal diversity at a family level, and a tendency toward increased diversity at other taxonomical levels was also found in nonobese individuals.^60^ More importantly, obese patients exhibited different fungal compositions compared with people with normal BMI. Among these, obese subjects had a higher amount of fungi belonging to the *Ascomycota* phylum, *Saccharomyces* and *Tremellomycetes* classes, and *Dipodascaeae* and *Sachharomycetaceae* families. Moreover, increased *Candida*, *Nakaseomyces*, and *Penicillium* populations were observed in obese individuals^11–13^, and *Candida parapsilosis* was recently identified as a critical commensal fungus related to diet-induced obesity in mice.^61^ Additionally, obese individuals display higher yeast counts.^13^ On the other hand, the number of fungi belonging to the *Zygomycota* phylum and *Mucor* genus was decreased in the obese population compared with individuals with normal weight.^60^ Notably, this negative correlation was reversed after weight loss.

The study of the association of gut fungi composition with anthropometrical and metabolic parameters related to obesity showed that the relative abundance of some fungi was linked to adiposity and related metabolic disorders, including insulin resistance, dyslipidemia, blood pressure, and inflammatory activity.^60^ Among the most important, the relative abundance of fungi belonging to the *Ascomycota* phylum, classes *Saccharomycetes*, *Tremellomycetes*, and *Cystobasidiomycetes*, families *Erythrobasidiaceae* and *Dipodascaceae*, and genera *Aspergillus, Eurotium* and *Rhodotorula*, increased with the occurrence of metabolic abnormalities. Contrary to that, the relative abundance of fungi belonging to the phylum *Zygomycota*, classes *Agaricomycetes* and *Eurotiomycetes*, families *Mucoraceae*, *Nectriaceae*, *Ceratocystidaceae*, *Corticiaceae*, *Debariomycetaceae* and *Hypocraceae* and genera *Mucor, Penicillium, Monilliela* and *Ceratocystis* were correlated with protection from these metabolic disorders. However, even if there are some associations between gut mycobiota and obesity, it is still unclear whether changes in fungal intestinal structure contribute to obesity development or are just an effect of different dietary habits leading to excessive weight, such as high carbon or high-fat consumption. Mice fed with a diet rich in fats exhibited a higher abundance of *Alternaria*, *Saccharomyces*, *Septoriella*, and *Tilletiopsis* genera, as well as *Saccharomyces cerevisiae* and *Tilletiopsis washingtonensis* species.^62^

The finding that obesity is associated with systemic inflammation, and the suggestion that the obesity-related fungal intestinal composition has a proinflammatory effect^63^, is particularly interesting in the context of the NAFLD. One of the questions that is still unanswered is whether altered microbiota contributes to liver inflammation in NAFLD. Research shows that chronic inflammatory disorders are associated with the disruption of the intestinal epithelial barrier, which, when intact, protects the host from bacterial invasion. Moreover, the gut microbiome may play a critical role in maintaining this intestinal epithelial barrier.^64^ The proinflammatory effect associated with obesity-related mycobiota might be one of the causes of the leaky intestinal barrier, further leading to liver inflammation.

The research devoted to gut fungal microbiota in NAFLD subjects showed that the relative abundances of *Talaromyces*, *Paraphaeosphaeria*, *Lycoperdon*, *Curvularia*, *Phialemoniopsis*, *Paraboeremia*, *Sarcinomyces*, *Cladophialophora*, and *Sordaria* were higher in patients with NAFLD. In contrast, the abundances of *Leptosphaeria*, *Pseudopithomyces*, and *Fusicolla* were decreased.^65^ What is more, patients with NAFLD displayed more co-occurring fungal intrakingdom correlations. The richness and evenness of fungal microbiota were increased in patients with nonalcoholic steatohepatitis (NASH) compared with patients without NASH. The same trend was observed for patients with fibrosis.

Furthermore, several fungi genera exhibited higher abundance in liver injury, lipid metabolism, and the development of NAFLD, e.g., *Paramycosphaerella*, *Fusicolla*, *Arthrinium*, *Triparticalcar*, *Trichoderma*, and *Cladosporium* genera were significantly more abundant in patients with NASH; *Cladosporium*, *Staphylotrichum*, *Paecilomyces*, and *Thermomyces* fungi were increased in patients with significant fibrosis. On the other hand, *Pulvinula*, genera with decreased abundance in patients with significant fibrosis, was negatively correlated with the fibrosis stage, Alanine aminotransferase (ALT), and Aspartate aminotransferase (AST). Another study showed that patients with NAFLD and more severe disease stages have a specific composition of fecal fungi and an increased systemic immune response to *C. albicans*.^66^ Interestingly, these changes could be observed particularly in patients without obesity, underscoring the previously unreported aspects of the nonobese phenotype of NAFLD. Moreover, in a fecal microbiome-humanized mouse model of Western diet-induced steatohepatitis, treatment with antifungal amphotericin B reduced liver damage. This observation suggests that intestinal fungi may be an attractive therapeutic target to minimize NASH and NAFLD progression. Although, it should be pointed out that amphotericin B interacts with cholesterol^67^ and may affect Toll-like receptor (TLR) signaling^68^, which may contribute to its beneficial effects on NAFLD and NASH, taking into account that cholesterol and TLR signaling contribute to NASH and NASH fibrosis.

## Primary sclerosis cholangitis (PSC)

PCS is a chronic liver disease manifested by the biliary tree’s progressive sclerosis (scarring). It is associated with inflammation of the bile ducts that leads to periductal fibrosis, destruction of the bile ducts, and consequently, biliary cirrhosis and liver failure.^69^ Of notice, approximately 75% of patients with PSC also have inflammatory bowel disease (IBD), while only about 7%–8% of patients with IBD have PSC.^70^ On the one hand, the strong association of PSC with IBD seems to support the hypothesis that PSC has an autoimmune etiology. On the other hand, some authors indicate that PSC cannot be considered a classical autoimmune disease, as it occurs with a 2:1 male predominance and lacks a characteristic response to immunosuppressants.^71,72^ Interestingly, the changes in the gut microbiome, including fungi, might contribute to PSC etiology.

One of the general concepts of damaging bile ducts includes the leaky gut hypothesis, but there are also some other more detailed hints that suggest the role of fungi in the pathogenesis of primary sclerosis cholangitis. Genetic variants of CARD9, a protein involved in the innate immunity against fungi, cause susceptibility to PSC and IBD.^24^ Furthermore, the anti-*S. cerevisiae* antibodies (ASCA) are highly prevalent in subjects with PSC^73^, and the presence of fungi belonging to the *Candida* genus in bile was associated with reduced survival in PSC patients.^74^ Fungi belonging to *Candida* genus have the potential to induce Th17 response in T cells^75^, and increased Th17 levels have been observed in PSC patients and recently been suggested to be involved in PSC pathogenesis.^76^

Also, Lemoinne et al.^77^ indicated that patients with PSC displayed fungal gut dysbiosis at various levels. Although in their study, there were no differences in mycobiota alpha diversity (Shannon and Chao1 indexes) among healthy subjects, patients with IBD only, PSC and IBD, and PSC only, they showed that samples clustered according to groups by beta diversity analysis. Of notice, PSC subjects had an increased proportion of fungi belonging to the *Sordariomycetes* class, and *Exophiala* genus, and a decreased proportion of *S. cerevisiae*. The putative positive effect of *S. cerevisiae* on the biliary tracts seems to be supported by the fact that *S. cerevisiae* level was also decreased in patients with active IBD, and it was shown to have anti-inflammatory effects by producing cytokine interleukin 10.^78^ A similar study performed on the other cohort revealed increased levels of the genera *Candida* and *Humicola* (species level annotation suggests *H. grisea*) in PCS subjects.^79^ As *H. grisea*, recently reclassified as *Trichocladium griseum*, belongs to the *Sordiomycetes* class, this finding supports the results of Lemoinne et al.^77^ at increased taxonomic resolution.

Also, the negative impact of *Exophiala* on the biliary ducts seems to be supported by other studies. Fungi belonging to the *Exophiala* genus have been connected to infections known as phaeohyphomycosis in hosts with an impaired immune system. Although most of these infections affect the skin, *Exophiala* is also able to cause systemic infections.^80^ Particularly, one case report described a systemic infection by *Exophiala dermatitidis* mimicking PSC in a patient without immunodeficiency.^81^ Another case report found that *E. dermatitidis* infection leads to an end-stage liver disease characterized by cholestasis and dilatation of intrahepatic bile ducts.^82^

Moreover, Lemoinne et al. observed a relative increase in biodiversity, such as patients with PSC had a higher ITS2/16S diversity ratio than IBD-only patients, suggesting an increased fungi-to-bacteria diversity ratio. What is more, the gut microbiota of patients with PSC exhibited a strong disruption in the bacteria-fungi correlation network compared with patients with IBD and healthy subjects, indicating an alteration in the interkingdom crosstalk.

## Cirrhosis

Cirrhosis is a late stage of liver scarring caused by chronic liver injury.^83^ It may be caused by many diseases, e.g., hepatitis, fatty liver disease, and chronic alcohol consumption.^84^ Injury of the liver leads to the formation of scar tissue called fibrosis. As a result, the liver is progressively damaged and cannot function properly. In advanced stages, cirrhosis leads to a life-threatening condition. In fact, cirrhosis is a leading cause of death worldwide.^85^ Interestingly, intestinal infections are one of the leading causes of mortality.^86^

The relationship between intestinal infections and cirrhosis has been investigated mainly for bacteria. Nevertheless, the role of fungi has been gaining attention recently. One study showed that a combined bacterial-fungal dysbiosis metric, *Bacteroidetes*/*Ascomycota*, changes with cirrhosis severity.^87^ Moreover, a lower *Bacteroidetes*/*Ascomycota* ratio was associated with lower hospitalizations, and this metric could predict 90-day hospitalizations in patients with cirrhosis. Notably, this prediction was independent of disease severity, encephalopathy, and clinical biomarkers. Fungal diversity was decreased in subjects with cirrhosis compared to healthy controls and inversely correlated with a model for end-stage liver disease (MELD) score. Antibiotics lowered the bacterial and fungal diversity, with the exception of *Candida*, for which a higher abundance was observed. Omeprazole, a proton pump inhibitor, changed bacterial diversity but did not affect the fungal metrics. *C. albicans* has also been detected in about 20% of fecal samples of cirrhotic subjects during rifaximin treatment.^88^

Contrary to the abovementioned results, patients who developed cirrhosis due to chronic hepatitis B virus (HBV) infection had increased fungal abundance and fungal diversity compared with patients with chronic hepatitis B, HBV carriers, and healthy controls.^89^ Moreover, certain fungal species were detected in higher abundance in HBV-associated cirrhosis, such as *Aspergillus*, *Candida*, *Galactomyces*, *Saccharomyces*, and *Chaetomium*. The copies of target DNA for *C. albicans*, *C. parapsilosis*, and *C. krusei* were significantly increased in HBV-associated liver cirrhosis subjects compared to HBV carriers and healthy volunteers.^90^ What is more, HBV-associated cirrhosis patients had a significantly increased prevalence of *C. parapsilosis*, *C. glabrata*, *C. tropicalis*, and *S. cerevisiae* than healthy volunteers. The increased abundances of *Saccharomyces* and *Candida* in HBV-associated cirrhosis were also confirmed by other studies.^91,92^ Another study identified liver cirrhosis as an independent risk factor for esophageal candidiasis via multivariate logistic regression analysis.^93^ However, in our opinion, the question remains whether the cause was not misplaced with the consequence in this case, as it seems equally probable that esophageal candidiasis predisposes to liver cirrhosis, and this type of statistical analysis shows only correlations, not causality.

In light of the abovementioned research, it is worth mentioning that infection with *Candida* is one of the major threats for patients with cirrhosis, with a reported mortality rate of 55% associated with invasive candidiasis in patients with cirrhosis.^94^ The odds of mortality in cirrhosis patients having invasive candidiasis are 4.4 times higher than those without invasive candidiasis.

## Hepatocellular carcinoma

Hepatocellular carcinoma (HCC) is the most common form of liver cancer. It is also one of the leading causes of cancer-related deaths worldwide^95^, and its incidence is estimated to cross annually 1 million cases by 2025.^96^ The pathogenesis of HCC is multifactorial^97^, and multiple risk factors have been identified, with HBV, HCV, and NASH at the top of them. In general, the gut microbiome of HCC patients differed from that of healthy subjects, and what is more, the intestinal microbiome profile showed a correlation with different etiologies.^98,99^ However, the contribution of gut fungi to this image has been poorly investigated, concentrated on the role of the fungal metabolites in the development of HCC.

For example, aflatoxin B1 (AFB1), a metabolite of several species from the *Aspergillus* genus, e.g., *Aspergillus flavus* and *Aspergillus parasiticus*, has been shown to contribute to HCC development.^100,101^ Aflatoxin-B1 is converted by members of the cytochrome p450 family into highly reactive intermediates that bind to DNA in hepatic cells, forming promutagenic adducts. The adducts interact with guanine bases of DNA and cause an arginine to serine mutation at codon 249 of the *Tp53* tumor suppressor gene, leading to hepatocarcinogenesis.^102,103^ There is also a high correlation between HBV, another risk factor for HCC, and aflatoxin B1, as both of these factors are frequent in populations with a high incidence of HCC. For example, a prospective China-based population study revealed that the interaction between AFB1 and HBV infection increased the risk of HCC 60-fold.^104^ These findings may suggest that HBV and aflatoxin B1 interact, increasing the risk of HCC. The observations showed that patients chronically infected with HBV have higher concentrations of AFB1 adducts than uninfected individuals.^105,106^ The binding of AFB1 metabolites to DNA may increase the risk of integration of viral DNA and malignant transformation as a result.^107^ Integrating the HBV x gene into the host cells inhibits nuclear excision repair responsible for removing AFB1-DNA adducts, favoring the persistence of mutations.^108,109^ HBV x, in cooperation with p53, may also contribute to uncontrolled cell proliferation.^108,110^ Another possibility is that increased hepatocyte necrosis and proliferation caused by HBV infection increase the likelihood of AFB1 mutations, including 249ser, and the subsequent clonal expansion of cells containing these mutations.^111^

At the level of mycobiome, one research has shown that patients with HCC had significantly decreased gut mycobiome diversity and increased abundance of *C. albicans* compared to those with liver cirrhosis.^112^ At the family level, patients with HCC showed a decreased abundance of *Myxotrichaceae*, *Debaryomycetaceae*, *Trichomonascaceae*, and *Saccharomycetaceae*, and an increased abundance of *Saccharomycetales fam Incertae sedis*. At the class level, a lower abundance of *Leotiomycetes* was observed than in liver cirrhosis, and at the genus level, *Kazachstania*, *Debaryomyces*, *Xeromyces*, *Amorphotheca*, and *Blastobotrys* were less enriched, whereas *Candida* was overrepresented. Interestingly, mice fed with *C. albicans* exhibited increased tumor size and body weight. This observation may suggest that abnormal colonization by *C. albicans* contributes to the growth of liver tumors.

Moreover, further analysis revealed 46 upregulated and 70 downregulated metabolites in the blood plasma of *C. albicans*-gavaged mice compared to control groups, suggesting that *C. albicans* can also change plasma metabolism, involving metabolites and the corresponding signaling pathway. Among the top upregulated metabolites in HCC subjects were L-carnitine and L-acetylcarnitine, which concentrations were previously reported by other studies to differentiate HCC patients from those with liver diseases or health controls.^113,114^ The link between L-carnitine and L-acetylcarnitine and HCC is not clear. On the one hand, an increase in these metabolites may be the result of the boosted requirement of energy consumption in HCC patients, which results in long-chain acylcarnitines accumulation and activation of the carnitine shuttle system for oxidation of long-chain fatty acids to supply more usable energy.^112^ On the other hand, it has been shown that an administration of L-carnitine in mice can prevent the progression of nonalcoholic steatohepatitis and inhibit liver carcinogenesis by suppressing oxidative stress and inflammation in the liver.^115^

Moreover, experiments also showed that NLRP6, which regulates host defense against microbes^116,117^, is necessary for promoting HCC caused by the abnormal colonization of *C. albicans*. This finding could be essential for providing new targets for the treatment of HCC.

## Fungi as probiotics

*S. cerevisiae* var. *boulardii* has been used for treating various gut-related diseases.^118^ It has been shown to change the gut microbiome and, more importantly, attenuate acute liver injury, hepatic steatosis, and low-grade inflammation.^119,120^ For example, *Saccharomyces boulardii* is used in clinical practice as a probiotic, presenting therapeutically important pathways for using gut mycobiota in NAFLD prevention and treatment. Research shows that in mice with diet-induced diabetes and obesity, oral administration of *S. boulardii* daily for four weeks resulted in body weight, fat mass, hepatic steatosis, and inflammation reduction. This was accompanied by increased cecum weight and changes in gut microbiota composition at the phylum, family, and genus levels.^120^ In rats fed with a high-fat diet, oral gavage of *S. boulardii* (7.5×109 CFU/kg/d) for eight weeks resulted in reductions of body weight, liver mass, liver index, hepatic steatosis, endotoxemia, and inflammation. *S. boulardii* can also adjust the proportion of *Escherichia coli* and *Bacteroides* in the intestine of NAFLD rats.^121^

*Agaricus bisporus* ^122–124^ and *Pleurotus ostreatus* ^125^, two types of macrofungi, also possess hepato-protective activity. Polysaccharide components of these fungi, among others, lowered ALT and AST concentrations in serum in a dose-dependent manner, reduced hepatocellular degeneration, necrosis, inflammatory infiltration, and CCl_4_-induced liver injury, as well as enhanced the antioxidant status, and improved lipid metabolism.

Other possible beneficial fungi such as *Hanseniaspora osmophila* and *Lachancea thermotolerans* ^126^ have not been validated in liver diseases. However, it should also be noted that *Clostridium difficile* colitis and neutropenic patients had *S. cerevisiae* fungemia after using *S. boulardii* as a probiotic.^127,128^ This observation shows that potential fungal probiotics should be treated with caution, especially in the case of immunocompromised patients.

## Conclusions

The research provides ample evidence that the intestinal mycobiota may impact the functioning of the liver and may play an essential role in the pathogenesis of the liver disease. Even though it may not always be clear whether the changes in the intestinal mycobiota are the cause of lesions in the liver or the result of other factors that damage the liver, there is no doubt that a balanced gut mycobiome contributes to the maintenance of the host immune homeostasis. Research into intestinal mycobiota and knowledge of the interplay between mycobiota and liver promote a better understanding of the pathogenesis of liver disease and thus allow for the development of new pathways for diagnosis, treatment, and prognosis for patients with liver disease.

From the role of the intestinal barrier, through specific metabolic pathways and mediators, to interactions between bacteria and fungi, the research on the intestinal microbiome shows a complicated picture of the relationship between fungi living in the intestine and liver dysfunction. Given the mounting evidence of bilateral interaction between the gut and liver, there is also no doubt that intestinal mycobiota research is required. However, even more is needed to transfer the scientific findings on the ground of well-planned trials, which then have a chance to turn into effective therapies. It is worth mentioning here that when considering therapies targeting intestinal mycobiome, e.g., antifungal therapies, it would be important to define the specific sub-groups of patients that would mostly benefit from antifungal therapy given the diversity of mycobiome in the heterogeneous human population. This approach fits in with the assumptions of precision medicine – an up-and-coming and extensively developing field of research and healthcare.

Whether we would like to use mycobiome as a diagnostic instrument, prognostic marker, treatment tool, e.g., fecal transplant or probiotics, or direct object of therapy in the case of harmful fungal strains, to make this possible, more large-scale, longer-term, longitudinal studies are necessary. The planned research should also be accompanied by joint analyses on gut mycobiome with other methods, such as metagenomics and metabolomics, as only this kind of comprehensive approach has the potential to deliver as complete picture as possible of the interplay between microbiota and the host.

## List of abbreviations

ALD: Alcohol-associated liver disease
ALT: Alanine aminotransferase
AST: Aspartate aminotransferase
AUD: Alcohol use disorder
HCC: Hepatocellular carcinoma
IBD: Inflammatory bowel disease
IL: Interleukin
ITS: Internal transcribed spacer
NAFLD: Nonalcoholic fatty liver disease
NASH: Nonalcoholic steatohepatitis
PSC: Primary sclerosis cholangitis
